# Traditional Medicine for Treatment of Neurodegenerative Diseases

**DOI:** 10.1155/2014/169821

**Published:** 2014-06-29

**Authors:** Chun Fu Wu, Wenxia Zhou, Chuthamanee Suthisisang, Jing Yu Yang

**Affiliations:** ^1^Department of Pharmacology, Shenyang Pharmaceutical University, Shenyang 110016, China; ^2^Beijing Institute of Pharmacology and Toxicology, Beijing 100850, China; ^3^School of Pharmaceutical Science, Mahidol University, Nakhon Pathom 73170, Thailand

Neurodegenerative diseases (NDD), such as Alzheimer's disease (AD), Parkinson's disease (PD), and amyotrophic lateral sclerosis (ALS), are one of leading causes of death all over the world. Traditional medicine (TM), especially traditional Chinese medicine (TCM), usually having good clinical tolerability, is applied to treat NDD in China based on TCM or modern pharmacological theories. This issue on TM for treatment of NDD compiles 12 exciting manuscripts, most of which reported the efficacy and possible mechanisms of action of TM or active constituents on AD, PD, and ALS both in clinical and experimental levels.

The effects of TCM on AD both in clinical and experimental levels are addressed in ten manuscripts. Dr. P. Wang et al. performed a survey of TCM treatment for AD, including Chinese herbal compound prescriptions (Danggui-Shaoyao-San, Yokukansan, and Bushenhuatanyizhi instant granules) and Chinese materia medica ingredients, and confirmed the certain complementary cognitive benefits of TCM therapy. This paper is accompanied by other 8 manuscripts associated with TM or TCM.

For clinical study, Dr. W. Pan et al. evaluated the effects of Shen-Zhi-Ling oral liquid on the behavioral and psychological symptoms of dementia in 98 patients with AD. Moreover, Dr. L. An et al. reported an updated meta-analysis of placebo-controlled RCTs of Huperzine A on patients with AD and vascular dementia (VD).

For experimental study, Dr. Z. Zhang et al. reported the potential suppressing effects of a new type of* Ginkgo biloba* extract, GBE50, on activated microglia by inhibiting signal transduction through the NF-*κ*B p65 and p38 MAPK pathways, which suggest the potential role of GBE on neuroinflammation related AD. Dr. L.-B. Zou et al. studied that xanthoceraside, a triterpene extracted from the husks of* Xanthoceras sorbifolia* Bunge, ameliorates mitochondrial dysfunction contributing to the improvement of learning and memory impairment in mice with intracerebroventricular injection of A*β*1-42. Dr. L. Zhang and L. Li found that cornel iridoid glycoside (CIG), an ingredient extracted from a traditional Chinese herb* Cornus officinalis*, attenuated tau hyperphosphorylation at multiple AD-related sites by increasing the activity of protein phosphatase 2A (PP2A) in SK-N-SH cells. Dr. R. Pan et al. reported that the crude extract of* Polygala tenuifolia* improved memory function of the aged mice probably via its antioxidant properties and decreased the activities of MAO and AChE. Dr. S.-J. Song et al. developed a bioactivity-oriented screening platform based on a modified Ellman's method and HPLC-QTOF MS technique and rapidly screened and confirmed some active compounds of* Anemarrhena asphodeloides *Bge. Dr. W.-X. Zhou and Y.-X. Zhang reported that Danggui-Shaoyao-San, could alleviate cognitive dysfunction in female senescence-accelerated mouse prone 8 (SAMP8) via modulation of estradiol.

In addition to AD, two manuscripts reported the effects of TCM on PD and ALS, respectively. Dr. C.-F. Wu et al. reported that pseudoginsenoside-F11 (PF11), a component of* Panax quinquefolius* (American ginseng), markedly improved the locomotor, motor balance, coordination, and apomorphine-induced rotations in 6-OHDA lesioned rats, and suggested its potent anti-Parkinson's property through inhibiting free radical formation and stimulating endogenous antioxidant release. Dr. W. Pan et al. investigated the use of integrative therapies (vitamins, Chinese herb decoctions, Chinese herb compounds, massage therapy, and acupuncture) in patients with ALS in Shanghai, China.

As for the experimental model to evaluate the effect of TCM on NDD, Dr. KS Cho reviewed the successful application of* Drosophila* models, to evaluate the effects of SuHeXiang Wan,* Gardenia jasminoides* Ellis components, and* Gastrodia elata* Blume extract, celastrol as well as curcumin.

By compiling these papers, we hope to enrich our readers and researchers with respect to the recent advancement of traditional medicine therapy on NDD.

*Chun Fu Wu* *Chun Fu Wu* *Wenxia Zhou* *Wenxia Zhou* *Chuthamanee Suthisisang* *Chuthamanee Suthisisang* *Jing Yu Yang* *Jing Yu Yang*

